# Post-Transcriptional Up-Regulation of PDGF-C by HuR in Advanced and Stressed Breast Cancer

**DOI:** 10.3390/ijms151120306

**Published:** 2014-11-06

**Authors:** Nian-An Luo, Ya-Qi Qu, Guo-Dong Yang, Tao Wang, Ren-Li Li, Lin-Tao Jia, Rui Dong

**Affiliations:** 1 Department of General Surgery, Tangdu Hospital, Fourth Military Medical University, Xi’an 710032, China; E-Mails: lna2012@fmmu.edu.cn (N.-A.L.); 604829845@163.com (Y.-Q.Q.); liren_li_ok@126.com (R.-L.L.); 2 Department of General Surgery, 10 Hospital of People’s Liberation Army (PLA), Wuwei 733000, China; 3 Department of Biochemistry and Molecular Biology, Fourth Military Medical University, Xi’an 710032, China; E-Mails: guodong.yang029@gmail.com (G.-D.Y.); wangt78@fmmu.edu.cn (T.W.)

**Keywords:** breast neoplasms, HuR, mRNA stability, platelet-derived growth factor-C (PDGF-C)

## Abstract

Breast cancer is a heterogeneous disease characterized by multiple genetic alterations leading to the activation of growth factor signaling pathways that promote cell proliferation. Platelet-derived growth factor-C (PDGF-C) is overexpressed in various malignancies; however, the involvement of PDGF-C in breast cancers and the mechanisms underlying PDGF-C deregulation remain unclear. Here, we show that PDGF-C is overexpressed in clinical breast cancers and correlates with poor prognosis. PDGF-C up-regulation was mediated by the human embryonic lethal abnormal vision-like protein HuR, which stabilizes the PDGF-C transcript by binding to two predicted AU-rich elements (AREs) in the 3'-untranslated region (3'-UTR). HuR is up-regulated in hydrogen peroxide-treated or ultraviolet-irradiated breast cancer cells. Clinically, HuR levels are correlated with PDGF-C expression and histological grade or pathological tumor-node-metastasis (pTNM) stage. Our findings reveal a novel mechanism underlying HuR-mediated breast cancer progression, and suggest that HuR and PDGF-C are potential molecular candidates for targeted therapy of breast cancers.

## 1. Introduction

Growth factors and their downstream signaling pathways play important roles in the uncontrolled proliferation and apoptosis resistance characteristic of malignant cells [1,2,3]. Platelet-derived growth factor-C (PDGF-C) is a new member of the PDGF family that is expressed in epithelial cells, muscle, and neuronal progenitors [4]. Upon association with the homodimeric PDGF-C, the PDGF receptor (PDGFR) dimerizes and activates several canonical signaling pathways such as phosphoinositide-3-kinase Akt (PI3K/Akt), Ras mitogen-activated protein kinase (Ras/MAPK), and phospholipase C-γ/Ca^2+^ (PLC-γ/Ca^2+^), promoting cell survival, proliferation, and focal adhesion [4,5]. Aberrant expression of PDGF-C is implicated in various malignancies, in particular glioblastoma and Ewing family sarcoma [4,6,7]. PDGF-C contributes to tumorigenesis by acting directly on neoplastic cells or activating cancer stromal cells such as fibroblasts to facilitate tumor development [3,4,8]. However, the regulation of PDGF-C and the mechanisms underlying its up-regulation in carcinoma cells remain largely unknown.

The regulation of gene expression in eukaryotes occurs at multiple levels [9,10,11]. Post-transcriptional gene regulatory events, especially regulation of mRNA turnover, emerged as fundamental and effective means to alter the expression of functionally related genes [10,11]. A cohort of RNA-binding proteins stabilizes mRNAs by association with the AU-rich elements (AREs) in the 3'-untranslated region (3'-UTR) [11,12]. Among the ARE-binding proteins, the human embryonic lethal abnormal vision (ELAV)-like protein family consists of four members (Hel-N1/HuB, HuC, HuD, and HuR) that are well characterized [13]. HuR is expressed in many cell types, whereas the other three proteins are expressed in terminally differentiated tissues [12,13]. HuR binds to labile transcripts containing AREs, such as mRNAs for proto-oncogenes, cytokines, and cytokine-response genes in the nucleus. The HuR-mRNA complex is then transported to the cytoplasm, where the stabilized mRNA can be efficiently translated [14,15].

Breast cancers, which represent 23% of overall female cancers and cause 40,000 deaths each year in the USA alone, are characterized by gene signatures of growth factors such as PDGFs [7,16]. Transcriptional activation plays a crucial role in PDGF-C up-regulation in neoplastic cells; however, accumulating data suggest that the control of mRNA stability and translational efficiency is critically involved in regulating PDGF-C expression [4]. In the present study, we showed that high PDGF-C levels are positively correlated with the tumorigenic capacity of breast cancer cells and are associated with advanced stage clinical breast cancers. PDGF-C up-regulation in breast cancer cells was at least partially mediated by transcript stabilization by the mRNA binding protein HuR, which was concomitantly up-regulated in advanced breast cancers. The biological roles of HuR and PDGF-C up-regulation under stress conditions were also examined.

## 2. Results and Discussion

### 2.1. Results

#### 2.1.1. Correlation of Platelet-Derived Growth Factor-C (PDGF-C) Expression with Poor Prognosis of Breast Cancers

**Figure 1:**
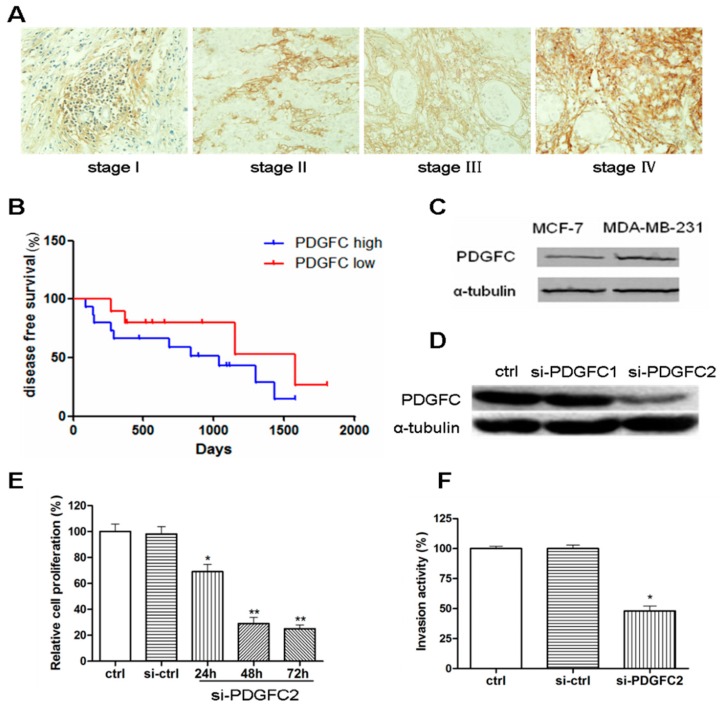
Platelet-derived growth factor-C (PDGF-C) is highly expressed in advanced breast cancers. (**A**) Representative immunohistochemical staining of PDGF-C in different pathological tumor-node-metastasis (pTNM) stage breast cancers (×400); (**B**) Patients were divided into high and low PDGF-C groups, and disease-free survival rates were plotted; (**C**,**D**) Western blot analysis of the lysates of the indicated breast cancer cell lines (**C**) or MDA-MB-231 cells transfected with the indicated siRNAs (**D**); (**E**) MTT [3-(4,5-dimethylthiazol-2-yl)-2,5-diphenyl-2H-tetrazolium bromide] assays were performed 48 h post-transfection of MDA-MB-231 cells with a control or PDGF-C-targeted siRNA; and (**F**) Relative invasion activities compared with untransfected (ctrl) cells were determined by counting the numbers of invaded cells in a Transwell matrigel assay. Data are represented as the mean ± standard deviation (SD) of three independent assays for **E** and **F**. * *p* < 0.05, ** *p* < 0.01, compared with the control group.

The expression of PDGF-C was examined in clinical breast cancer specimens of different pathological tumor-node-metastasis (pTNM) stages (Table 1). PDGF-C was detected in 49 of 81 (60.5%) breast cancer cases, and significantly increased PDGF-C levels were observed in high stage breast cancers (Figure 1A). The expression of PDGF-C was significantly correlated with histological grade (*p* = 0.021) and pTNM stage (*p* = 0.023) (Table 2). Patients were grouped according to the level of PDGF-C expression, as determined by immunohistochemical staining, which showed that PDGF-C-high breast carcinomas were associated with short disease-free survival compared with PDGF-C-low or -negative breast cancers (Figure 1B). PDGF-C expression was higher in the tumorigenic and invasive breast cancer cell line MDA-MB-231 than in the non-invasive cell line MCF-7 (Figure 1C). Knockdown of PDGF-C inhibited the proliferation and invasiveness of MDA-MB-231 cells (Figure 1D–F). Taken together, these results suggest that PDGF-C expression is correlated with the malignant phenotype and poor prognosis of breast cancers.

**Table 1:** Patient characteristics.

Parameter	Characteristics	No. of Patients (%)
**Age**	≤45	35 (43.2)
	>45	46 (56.8)
**Tumor Size**	≤3 cm	54 (66.7)
	>3 cm	27 (33.3)
**Histological Grade**	G1	26 (32.1)
	G2	45 (55.6)
	G3	10 (12.3)
**Pathological Tumor-Node-Metastasis (PTNM) Staging**	I	21 (25.9)
	II	39 (48.2)
	III	15 (18.5)
	IV	6 (7.4)
**Histological Type**	Ductal	66 (81.5)
	Lobular	11 (13.6)
	Others	4 (4.9)
**Lymph Node Metastasis**	0	41 (50.6)
	<4	16 (19.8)
	≥4	24 (29.6)
**HER**	Positive	56 (69.1)
	Negative	25 (30.9)
**Estrogen receptor (ER)**	Positive	35 (43.2)
	Negative	46 (56.8)
**Progesterone receptor (PR)**	Positive	37 (45.7)
	Negative	44 (54.3)

#### 2.1.2. Coordinated Expression of HuR and PDGF-C in Breast Cancers

**Figure 2:**
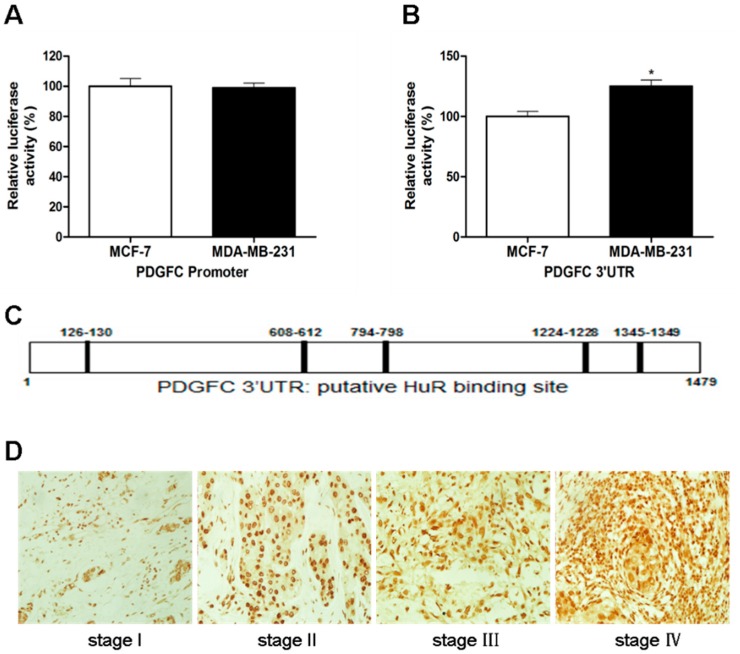
Correlation of PDGF-C and HuR expression in breast cancers. (**A**,**B**) The luciferase reporter construct of the PDGF-C promoter (**A**) or 3'-UTR (**B**) was generated and introduced into the indicated breast cancer cells. Cellular luciferase activity was measured and plotted as relative activity compared to MCF-7 cells; (**C**) Predicted HuR-binding sites in the 3'-UTR of PDGF-C; and (**D**) Representative immunohistochemical staining of HuR in different pTNM stage breast cancers (×400). Data are represented as the mean ± SD of three independent assays for **A** and **B**. * *p* < 0.05.

To elucidate the mechanism underlying the up-regulation of PDGF-C in advanced breast cancers, a luciferase reporter construct containing the PDGF-C promoter was generated and introduced into breast cancer cells with a distinct invasive potential. No significant differences in the activity of the PDGF-C promoter were detected between MCF-7 and MDA-MB-231 cells (Figure 2A), which was in contrast to a significantly higher stability of luciferase transcripts flanked by the PDGF-C 3'-UTR in MDA-MB-231 cells than in MCF-7 cells (Figure 2B). These data indicate that PDGF-C may be regulated at the post-transcriptional level in breast cancer cells.

**Table 2:** Relative of PDGF-C expression with clinicopathological characteristics and HuR expression.

Parameter		PDGF-C Expression		*p*
		Positive	Negative	
**Age**	≤45	15	20	0.251
	>45	26	20	
**Tumor Size**	≤3 cm	29	25	0.496
	>3 cm	12	15	
**Histological Grade**	G1	7	19	0.021
	G2	28	17	
	G3	6	4	
**pTNM Staging**	I	7	14	0.023
	II	20	19	
	III	9	6	
	IV	5	1	
**Histological Type**	Ductal	36	30	0.306
	Lobular	2	9	
	Others	3	1	
**Lymph Node Metastasis**	0	26	15	0.528
	<4	7	9	
	≥4	8	16	
**HER**	Positive	29	27	0.851
	Negative	12	13	
**ER**	Positive	24	11	0.417
	Negative	17	29	
**PR**	Positive	11	26	0.0853
	Negative	30	14	
**HuR**	Positive	36	21	0.029
	Negative	5	19	

The RNA-binding protein HuR binds to and stabilizes specific mRNAs and is thus implicated in diverse pathophysiological processes [12]. To determine whether PDGF-C is post-transcriptionally regulated by HuR, we first predicted the HuR-binding sites on the PDGF-C mRNA, and found multiple putative HuR-binding sites on the transcript (Figure 2C). Consistently, HuR was expressed at high level in advanced breast cancers (Figure 2D). The expression of PDGF-C was significantly correlated with histological grade (*p* = 0.021), pTNM stage (*p* = 0.023), and HuR (*p* = 0.029). Other clinicopathological factors examined showed no significant correlations with PDGF-C expression (Table 2). Therefore, high PDGF-C levels were associated with the up-regulation of HuR in advanced breast carcinomas.

#### 2.1.3. Direct Targeting and Stabilization of PDGF-C Transcripts by HuR in Breast Cancers

**Figure 3:**
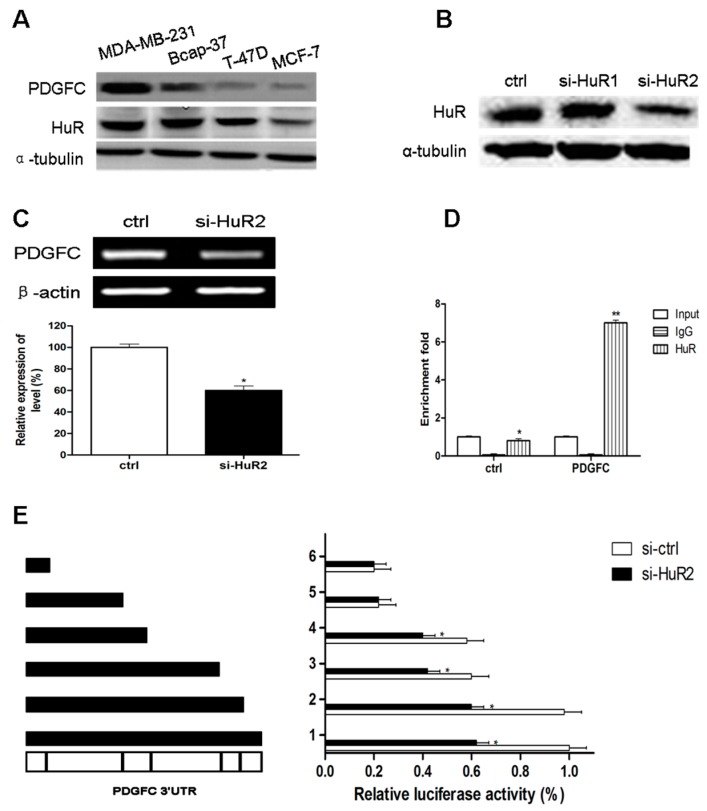
HuR binds the 3'-UTR of PDGF-C mRNA and stabilizes the mRNA transcript**.** (**A**) Western blot analysis of lysates of the indicated cell lines; (**B**) Western blot analysis of lysates of MDA-MB-231 cells 48 h post-transfection with control or HuR-targeted siRNA; (**C**) RT-PCR assay of MDA-MB-231 cells 24 h post-transfection with control or HuR-targeted siRNA; (**D**) Enrichment of PDGF-C but not control mRNA after immunoprecipitation of the lysates of MDA-MB-231 cells using a HuR antibody; and (**E**) Schematic representation of full-length and truncated 3'-UTR of PDGF-C containing the predicted HuR-binding sites (**left**); and relative luciferase activity of MDA-MB-231 cells co-transfected with the aforementioned 3'-UTR constructs and control or HuR siRNA (**right**). Data are represented as the mean ± SD of three independent assays for (**C**–**E**). * *p* < 0.05, ** *p* < 0.01, compared with the control (**C**,**E**) or IgG group (**D**).

We next examined whether PDGF-C is directly up-regulated by HuR in neoplastic mammary cells. We found that PDGF-C levels were correlated with the expression of HuR in breast cancer cell lines with varied invasion potentials (Figure 3A) Knockdown of HuR downregulated PDGF-C in MDA-MB-231 cells (Figure 3B,C). An RNA-immunoprecipitation (IP) confirmed the direct association of HuR with the PDGF-C transcript (Figure 3D). Reporter plasmids consisting of the luciferase coding sequence flanked by intact or truncated 3'-UTR of PDGF-C were co-transfected with control or HuR siRNAs into MDA-MB-231 cells (Figure 3E). The results showed that the absence of either the second or fourth proximal putative HuR-binding site significantly decreased luciferase activity, suggesting decreased mRNA stability due to attenuated HuR binding (Figure 3E). Knockdown of HuR further inhibited luciferase activity in the 3'-UTR constructs encompassing at least one of the two putative HuR-binding sites, supporting the protective role of these sites possibly mediated by HuR binding and stabilization of the transcripts (Figure 3E). Taken together, these findings indicated that the PDGF-C transcript was stabilized by direct interaction with HuR in breast cancer cells.

#### 2.1.4. Stress-Induced HuR Regulation of PDGF-C in Breast Cancers

**Figure 4:**
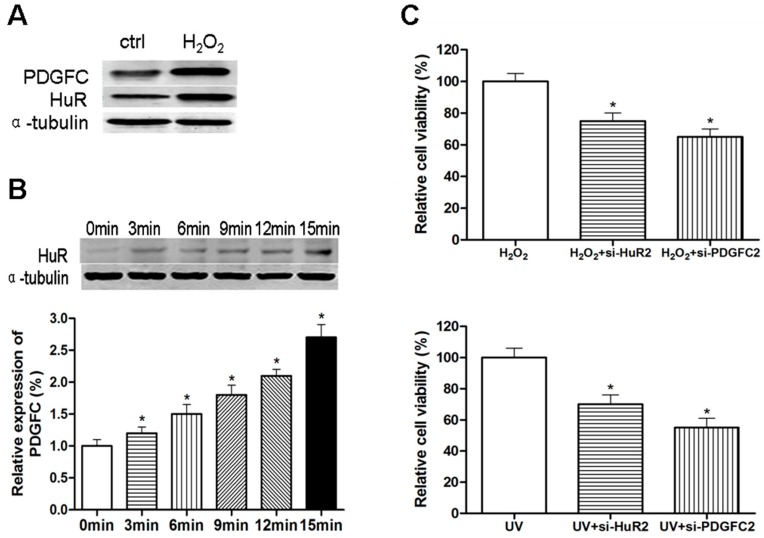
HuR up-regulates PDGF-C in response to stress. (**A**) Western blotting analysis of lysates of MCF-7 cells 24 h after treatment with H_2_O_2_ (800 µmol/L); (**B**) Western blotting for HuR (**upper panel**) and enzyme linked immunosorbent assay (ELISA) for PDGF-C production (**lower panel**) in MCF-7 cells 24 h after irradiation with UV for the indicated times; and (**C**) MTT assay was performed in MCF-7 cells treated with H_2_O_2_ (800 µmol/L) or UV irradiation and transfected with the indicated siRNAs. Data are represented as the mean ± SD of three independent assays for (**B**) and (**C**). * *p* < 0.05 compared with 0 min group (**B**, **lower panel**) or H_2_O_2_/UV treatment alone (**C**).

HuR expression plays a role in maintaining the malignant state of advanced carcinomas; however, induced expression of HuR, which has been reported frequently, may represent an adaptive response to diverse stress situations [17]. Consistent with the well-defined role of HuR in oxidative stress [18,19], treatment of MCF-7 cells with hydrogen peroxide (H_2_O_2_) up-regulated HuR, leading to the up-regulation of PDGF-C (Figure 4A). Similarly, a concomitant increase in HuR and PDGF-C was observed upon exposure of MCF-7 cells to ultraviolet (UV) irradiation (Figure 4B). Knockdown of HuR or PDGF-C sensitized MCF-7 cells to H_2_O_2_- or UV-triggered cell death (Figure 4C). Therefore, HuR-mediated up-regulation of PDGF-C is involved in the cellular protective responses to stress.

### 2.2. Discussion

Breast cancers are heterogeneous malignancies driven by germline or somatically accumulated genetic mutations [16]. Excessive signaling by steriod hormone and epidermal growth factor receptors is a characteristic of most breast cancers [2]. However, alternative inherent alterations also contribute to the occurrence and progression of breast cancers [2,16]. Here, we showed that platelet-derived growth factor-C (PDGF-C) is redundantly expressed in malignant cells, and its overexpression is correlated with advanced stage and poor prognosis of clinical breast cancers. Our findings are in agreement with previous reports that PDGF-C is overexpressed in various malignancies and plays essential regulatory roles in the tumor microenvironment [4,7,20,21]. Consistent with a pan-epithelial expression pattern of PDGF receptors [4], we found that PDGF-C plays a role in the accelerated proliferation of cultured breast cancer cells and confers resistance against stress-induced neoplastic cell death. However, our results do not exclude the effect of PDGF-C on tumor-associated macrophages, fibroblasts, and vascular endothelial cells, which expedite the progression of breast carcinomas [3,8,20,22].

The past decades have witnessed breakthroughs towards understanding of the post-transcriptional gene regulation machinery [10,11]. Unlike the degradation and translational suppression of messenger RNAs by microRNAs, HuR is among a cohort of RNA-binding proteins that regulate gene expression by stabilizing gene transcripts [10,11,12]. HuR is implicated in cell cycle and apoptosis regulation, angiogenesis, inflammation, and tumorigenesis by targeting numerous genes including cytokines and cyclins [23,24,25]. In the present study, we show that HuR binds to and stabilizes the PDGF-C transcript in advanced breast cancers, resulting in increased PDGF-C levels in neoplastic cells. Consistently, HuR expression was correlated with high levels of PDGF-C in late-stage breast cancers, suggesting that PDGF-C is among the key mediators of HuR-induced malignant transformation.

As a master regulator of gene transcript stability and splicing, HuR itself is tightly regulated at multiple levels including by transcriptional activation and post-translational modifications [17,26,27]. Nucleo-cytoplasmic shuttling plays a pivotal role in fine-tuning HuR activity under physiological or stress conditions [17,28,29]. The exact mechanisms underlying the regulation of HuR expression and cytoplasmic translocation remain unclear; however, various stress stimuli such as UV light, DNA damaging agents, or T cell activation promote the translocation of HuR to the cytoplasm, while AMP-activated kinase inhibits it [17,26,30,31]. Stress promotes the p38 MAPK-induced cytoplasmic translocation of HuR and the stability of ARE-containing mRNAs [32]. In the present study, we observed that H_2_O_2_ treatment or UV irradiation up-regulated HuR and PDGF-C expression in MCF-7 cells. Given that the effectiveness of standard cancer treatment is largely based on the induction of oxidative or DNA damage stresses [33,34,35], the role of HuR up-regulation in cancer resistance to clinical chemotherapy or radiotherapy merits further investigation. Collectively, our results shed light on the roles of HuR and PDGF-C in mammary carcinogenesis and indicate that these oncoproteins may serve as potential targets for the molecular classification and treatment of breast cancers.

## 3. Experimental Section

### 3.1. Cell Culture and Treatment

Human breast cancer MCF-7 and MDA-MB-231 cells were purchased from ATCC (Manassas, VA, USA). Cells were cultured in Dulbecco’s modified eagle’s medium (DMEM) supplemented with 10% fetal bovine serum (FBS) and maintained at 37 °C in a humidified incubator with 5% CO_2_. Where indicated, cells were treated with 800 µmol/L H_2_O_2_ for 24 h, irradiated with ultraviolet (UV) (30 J/m^2^) for 3 to 15 min, or transfected with Lipofectamine 2000 (Invitrogen, Carlsbad, CA, USA)-encapsulated siRNAs (Shanghai GenePharma Inc., Shanghai, China) before further examination. The PDGF-C- and HuR-targeted and control siRNA sequences were as follows: 5'-acugugucuacuaauggaatt-3' (sense) and 5'-uuccauuaguagacacagutt-3' (anti-sense) for si-PDGFC1; 5'-ggacuuagaagaucuauautt-3' (sense) and 5'-auauagaucuucuaagucctt-3' (anti-sense) for si-PDGFC2; 5'-uucuccgaacgugucacgutt-3' (sense) and 5'-acgugacacguucggagaatt-3' (anti-sense) as a control; 5'-ggaugaguuacgaagccugtt-3' (sense) and 5'-caggcuucguaacucaucctt-3' (anti-sense) for si-HuR1; 5'-ccaguuucaauggucauaatt-3' (sense) and 5'-uuaugaccauugaaacuggtt-3' (anti-sense) for si-HuR2; and 5'-uucuccgaacgugucacgutt-3' (sense) and 5'-acgugacacguucggagaatt-3' (anti-sense) as a control.

### 3.2. Reverse Transcriptase Polymerase Chain Reaction (PCR)

Total RNA was extracted using the Trizol reagent (Invitrogen) according to the manufacturer’s protocol. The reverse transcription reaction was performed using SuperScript™ II reverse transcriptase (Invitrogen) and oligo d(T) as a primer. The cDNAs were amplified using the following primers: 5'-gataccattttctggccaggttg-3' and 5'-tgcacacacagtcacactcctc-3' for PDGF-C; 5'-cccaaggccaaccgcgagaagat-3' and 5'-gtcccggccagccaggtccag-3' for β-actin.

### 3.3. Western Blot Analysis

Cells were washed in phosphate buffered saline (PBS) twice before protein extraction. Proteins were subjected to sodium dodecyl sulfate polyacrylamide gel electrophoresis (SDS/PAGE), transferred onto polyvinylidene difluoride (PVD) membranes, and analyzed by immunoblotting with antibodies against PDGF-C (GeneTex, Irvine, CA, USA), HuR (Proteintech, Chicago, IL, USA), or α-tubulin (Abcam, Cambridge, UK). Goat anti-rabbit and goat anti-mouse immunoglobulin horseradish peroxidase-linked F(ab)_2_ fragments (ZB-2305, Zhong Shan Jin Qiao, Beijing, China) were used as secondary antibodies.

### 3.4. MTT [3-(4,5-Dimethylthiazol-2-yl)-2,5-diphenyl-2H-tetrazolium bromide] Assay

Cells (3000/well) were seeded in 96-well plates in complete medium 24 h prior to transfection. Where indicated, H_2_O_2_ treatment or UV irradiation was performed 24 h post-transfection. After another 24 h, MTT [3-(4,5-dimethylthiazol-2-yl)-2,5-diphenyl-2*H*-tetrazolium bromide] in 100 μL of fresh serum-free medium was added to reach a final concentration of 0.5 g/L, followed by continued incubation at 37 °C for 4 h. The MTT-containing medium was then removed by aspiration and 50 μL of dimethyl sulfoxide (DMSO) was added. After incubation at 37 °C for a further 10 min, absorbance was measured at a wavelength of 490 nm in a plate reader.

### 3.5. Cell Invasion Assay

Cell invasion assay was performed in a 24-well Transwell (Corning, New York, NY, USA) on a polycarbonate filter pre-coated with 30 μg of Matrigel (BD Biosciences, Franklin Lakes, NJ, USA). 2 × 10^4^ cells suspended in 0.2 mL of serum-free medium were added to the upper well of the chamber, and 600 μL of medium supplemented with 10% FBS was added to the lower well. After incubation at 37 °C in 5% CO_2_ for 24 h, the cells on the upper chamber were removed by a cotton swab. Invaded cells on the bottom of the membranes were fixed with methanol and stained with 0.1% crystal violet solution. Cells were photographed under the microscope, and the cell numbers were counted by Image-Pro Plus 6.0 software (Media Cybernetics, Silver Springs, MD, USA) in five randomly selected fields.3.6. RNA Immunoprecipitation.

Cells were lysed in buffer containing 0.5% NP40, 0.5% Na Deoxycholate, 300 U/mL Superase Inhibitor (Ambion, Austin, TX, USA), and protease inhibitor (pH 7.9). Samples were treated with 30 U of Turbo DNase (Ambion) and incubated 15 min at 37 °C. After centrifuging for 5 min at 1350× *g* at 4 °C, 10% of the supernatant was saved as input, and the rest was subjected to immunoprecipitation at 4 °C overnight using 100 μL of Dynabeads Protein G conjugated with anti-HuR antibody (Proteintech) or IgG. Beads were washed three times at 4 °C with PBS supplemented with 1% NP40, 0.5% Na Deoxycholate, additional 300 mM NaCl, and 1:200 superase inhibitor. The immunocomplexes were eluted from the beads by the addition of 100 μL extraction buffer (50 mM Tris–HCl, pH 8.0, 10 mM EDTA, and 1.3% SDS) plus Superase inhibitor and incubated for 30 min at 65 °C. RNA was extracted from input samples and immunoprecipitates using Trizol reagent (Invitrogen). PCR was performed to amplify the cDNA of PDGF-C and GAPDH (ctrl) as described above in “Reverse transcriptase polymerase chain reaction (PCR)”. The results were normalized relative to the input control.

### 3.6. Luciferase Reporter Assays

For PDGF-C promoter activity assay, the reported promoter region of PDGF-C was amplified by genomic PCR from MCF-7 cells using the following primers: 5'-atgctagccctgaacacaagccacaaga-3' and 5'-cgctcgagttgttgctggaaaactggaa-3' [36]. The resulting 1.3 kb fragment was ligated into the NheI/XhoI sites of pGL3-enhancer vector and introduced into MCF-7 and MDA-MB-231 cells. Forty-eight hours after transfection, luciferase activity was assayed using the Dual-Luciferase Reporter Assay System (Promega, Madison, WI, USA) and a luminometer (Glomax 20/20, Promega).

For assaying HuR binding and regulation of PDGF-C transcript, the full-length and truncated 3'-UTR of mature PDGF-C mRNA were obtained by RT-PCR and inserted into the XbaI site downstream of the firefly luciferase gene in the pGL3-Promoter vector (Promega). The primer sequences were as follows: Shared upstream primer, 5'-tttctagaccgcatcaccaccagcagctc-3', and downstream primers, 5'-tttctagacctgaagatgaaaggtccttg-3', 5'-tttctagaggcagaaattttaataagaa-3', 5'-tttctagaacctttggtaaaaatagatata-3', 5'-tttctagaaggatgtgttcctactgcaca-3', 5'-tttctagatgccagagtacaataagtgaac-3', 5'-tttctagatccccaaaatagccacattc-3' for truncated variants 1–5 and full-length 3'-UTR, respectively. MDA-MB-231 cells were co-transfected with reporter constructs, an internal control vector (pGL4.73), and synthetic control or HuR-targeted siRNAs. Forty-eight hours after transfection, luciferase activity was measured using the Dual-Luciferase Reporter Assay System (Promega) and a luminometer (Glomax 20/20, Promega), and normalized to the activity of Renilla luciferase driven by a constitutively expressed promoter in the phRL vector. Basal promoter activity was expressed as the fold change relative to the activity observed with the basic pGL3 vector alone.

### 3.7. Enzyme Linked Immunosorbent Assay (ELISA) for PDGF-C

The total proteins of cultured MCF-7 cells were extracted using NE-PER Cytoplasmic Extraction Reagents (Pierce, Rockford, IL, USA) according to the manufacturer’s protocol, and protein concentration was determined using a Bio-Rad Protein Assay kit (Bio-Rad Laboratories, Hercules, CA, USA). PDGF-C levels were measured using an ELISA kit (Yanxin Biological Technology, Shanghai, China) according to the manufacturer’s instructions. The protein concentrations of PDGF-C were normalized and expressed as pictograms per milligram of total cellular protein.

### 3.8. Clinical Sample Collection

A total of 81 primary breast cancer patients were enrolled between January 2012 and February 2013 in Tangdu Hospital of the Fourth Military Medical University (Xi’an) in China. The clinical characteristics of patients were obtained from hospital records. Sample collection was approved by the Ethics Committee of the Fourth Military Medical University.

### 3.9. Immunohistochemistry

Formalin-fixed breast cancer samples were embedded in paraffin. Serial 4 μm sections were obtained using a Leica microtome. The sections were then deparaffinized in xylene and rehydrated in a descending ethanol series. Tissue antigen retrieval was performed using 0.01 mol/L Na-citrate buffer (pH 6.0) in a steamer at 100 °C for 15 min. The sections were then incubated in 0.3% H_2_O_2_ for 10 min to remove endogenous tissue peroxidase activity. After washing with PBS, nonspecific tissue binding sites were blocked for 20 min at room temperature with 10% horse serum. The tissue sections were then incubated overnight with a rabbit anti-HuR antibody (1:100; Proteintech) or a rabbit anti-PDGF-C antibody (1:200, GeneTex). The slides were washed with PBS and incubated for 1 h with biotin-labeled anti-rabbit secondary antibodies (1:200 in phosphate-buffered saline containing 1% normal horse serum) for 45 min. The tissue sections were incubated with the avidin-biotinylated reagent (Gene Tech, Shanghai, China) for 1 h. Antibody binding was visualized using III Detection System/Mo & Rb (Gene Tech) at 37 °C and then lightly counterstained with Mayer’s hematoxylin. As a positive control for PDGF-C expression, we used a human breast carcinoma cell line known to overexpress PDGF-C. When more than 10% of the cancer cells showed cytoplasmic staining for PDGF-C, the tumor was judged as positive for PDGF-C expression. We observed normal colon glands as a positive control for HuR staining.

The percentage of positive cells and intensity of staining were measured as indexes of HuR staining status. The percentage of positive cells was scored as follows: Zero (0% positive cells), 1 (<25% positive cells), 2 (25% to <50% positive cells), 3 (50% to <75% positive cells), and 4 (>75% positive cells). The intensity of cytoplasmic and nuclear staining was scored as follows: Zero (negative staining), 1 (weak staining), 2 (moderate staining), and 3 (strong staining). For the immunoreactivity score, values from 0 to 12 were multiplied by the positive cell score and intensity of staining score. Cases were classified as having negative or weak expression when the immunoreactivity score was 0 to 6; a score from 7 to 12 was regarded as strong expression.

### 3.10. Statistical Analysis

All statistical analyses were performed using SPSS 13.0 software (SPSS, Chicago, IL, USA). The association between the staining index and other clinicopathological variables was evaluated by the chi-squared test. A probability value below 0.05 was considered as statistically significant.

## 4. Conclusions

In the present study, we showed that PDGF-C expression was positively correlated with late-stage clinical breast cancers. PDGF-C was up-regulated at the post-transcriptional level by the human embryonic lethal abnormal vision (ELAV)-like protein HuR, which stabilizes mRNA transcripts via association with the 3'-UTR of target genes. HuR was induced by H_2_O_2_ treatment or UV irradiation of breast cancer cells. Clinically, HuR levels were correlated with PDGF-C expression and histological grade or pTNM stage. These findings reveal a novel mechanism underlying the role of HuR in breast cancer progression and suggest that HuR and PDGF-C could serve as molecular targets for the treatment of breast cancers.
